# The combined signatures of the tumour microenvironment and nucleotide metabolism-related genes provide a prognostic and therapeutic biomarker for gastric cancer

**DOI:** 10.1038/s41598-023-33213-z

**Published:** 2023-04-24

**Authors:** Jifeng Liu, Lei Zhong, Dawei Deng, Yunshu Zhang, Qihang Yuan, Dong Shang

**Affiliations:** 1 Department of General Surgery, The First Affiliated Hospital of Dalian Medical University, Dalian, Liaoning China; 2 Department of Hepato-Biliary-Pancreas, Affiliated Hospital of North Sichuan Medical College, Nanchong, China; 3 Department of Traditional Medicine, The First Affiliated Hospital of Dalian Medical University, Dalian, Liaoning China

**Keywords:** Cancer, Computational biology and bioinformatics, Molecular biology, Biomarkers, Gastroenterology, Molecular medicine, Oncology

## Abstract

The tumour microenvironment (TME) is vital to tumour development and influences the immunotherapy response. Abnormal nucleotide metabolism (NM) not only promotes tumour cell proliferation but also inhibits immune responses in the TME. Therefore, this study aimed to determine whether the combined signatures of NM and the TME could better predict the prognosis and treatment response in gastric cancer (GC). 97 NM-related genes and 22 TME cells were evaluated in TCGA-STAD samples, and predictive NM and TME characteristics were determined. Subsequent correlation analysis and single-cell data analysis illustrated a link between NM scores and TME cells. Thereafter, NM and TME characteristics were combined to construct an NM-TME classifier. Patients in the NMlow/TMEhigh group exhibited better clinical outcomes and treatment responses, which could be attributed to the differences in immune cell infiltration, immune checkpoint genes, tumour somatic mutations, immunophenoscore, immunotherapy response rate and proteomap. Additionally, the NMhigh/TMElow group benefited more from Imatinib, Midostaurin and Linsitinib, while patients in the NMlow/TMEhigh group benefited more from Paclitaxel, Methotrexate and Camptothecin. Finally, a highly reliable nomogram was developed. In conclusion, the NM-TME classifier demonstrated a pretreatment predictive value for prognosis and therapeutic responses, which may offer novel strategies for strategizing patients with optimal therapies.

## Introduction

Gastric cancer (GC) is the third leading cause of cancer-related mortality and the fifth most frequently diagnosed cancer worldwide^1^. Patients with GC are typically diagnosed at an advanced stage owing to the absence of early symptoms^2^. Despite advances in chemotherapeutic regimens for advanced GC, the efficacy of treatments remains poor, with overall survival (OS) rate of less than 2 years^3,4^. Thus, targeted therapy is a research hotspot for the development of the treatment for GC. Despite the development of several targeted medications recently, the overall results remain dismal^5^. Immunotherapy provides GC sufferers with more treatment options and offers hope for the disease's treatment. Although immunotherapy offers tremendous benefits to patients with GC, there are significant differences in sensitivity to immunotherapy among patients^6^. As a result, it is critical to develop appropriate biomarkers for the prognosis prediction and tailored treatment of patients with GC.

Increasing evidence suggests that the tumour microenvironment (TME) is critical to tumour development, progression and therapeutic resistance^7–11^. The presence of multiple cell types in the TME has been demonstrated to be essential for the anti-tumour immune response. Therefore, elucidating the cellular composition may not only provide prognostic information but also suggest the potential efficacy of immunotherapy^9,12,13^. Furthermore, transcriptomics data can be used for the large-scale investigation of the immunological landscape^14^. In the current study, we used the ‘CIBERSORT’ algorithm, which is considered the most reliable approach available and has already been used for immunoscore model creation in various cancer types^8,15,16^, to improve early detection and prognosis prediction in cancer. Nucleotides, a type of biological information macromolecule, serve primarily as the precursors for nucleic acid synthesis, thereby promoting cell proliferation^17^. Notably, nucleotide metabolism (NM) is the last and the most crucial link in malignant cell replication. Tumour cells utilise NM to synthesise DNA and RNA and consequently contribute to uncontrolled cell proliferation^18,19^. Recently, researchers have affirmed that abnormal NM enhances the growth of tumours and suppresses the normal immune responses in the TME^20^. For instance, altering the equilibrium of nucleotide pools can result in mutations that alter antigen presentation and, as a result, the immune response against the tumour^21,22^. Therefore, there exists a strong association between TME and NM, with both having a significant impact on the development of tumours and immunotherapy.

The expansion of study on NM and TME enhances our awareness of the significance of NM-related TME in cancer patient prognosis and treatment. Nonetheless, to the best of our knowledge, no combined study of NM and TME cells has been performed to predict the prognosis and immunotherapeutic response in patients with GC. In the current study, we, therefore, sought to methodically develop an NM-TME signature for the prognosis and therapeutic response prediction of patients with GC by integrating NM characteristics and TME cells. The combined signature constructed herein could better reflect the role of the TME in tumour prognosis and treatment than traditional multi-gene prognostic signatures. Although the combination signature may be more complicated, genome sequencing analysis for cancer genomic research and clinical applications is speculated to become more prevalent and advanced as sequencing costs continue to drop and computing resources continue to grow^23^. Thus, by quantifying specific immune cells and nucleotide metabolism-related genes (NMRGs) using sequencing technology and related algorithms, clinicians can effectively predict patient prognosis and guide personalised treatment based on this NM-TME signature. Meanwhile, our research may help to improve our understanding of tumour-specific biology based on an integrated manner of NM-related TME, which has significant clinical disease management ramifications.

## Methods

### Data collection

RNA-sequencing (RNA-seq) and the matched clinical characteristics of the TCGA-STAD cohort were obtained from The Cancer Genome Atlas (TCGA) database. Additionally, RNA-seq and clinical data of the GSE84437 cohort, comprising 433 GC samples, were downloaded from the Gene Expression Omnibus (GEO) database as a validation set^24^. Log transformed expression data from raw hybridisation arrays were downloaded and normalised using robust multi-array averaging^25^.

A total of 97 NMRGs were obtained from the Molecular Signatures Database^26^ (Supplementary Table S1). For TME cells, we employed CIBERSORT, a deconvolution algorithm^27^, to determine the relative proportions of 22 different types of immune cells. CIBERSORT enrichment values were used to indicate the quantity of each TME cell type in each tumour sample across all cohorts.

### Untargeted metabolomic strategies

A total of 33 patients with GC and 27 healthy volunteers were selected and enrolled from the First Affiliated Hospital of Dalian Medical University. The First Affiliated Hospital of Dalian Medical University’s institutional ethics committee approved this study. All included patients and healthy volunteers provided consent to the use of their blood samples for research by signing a written consent form. All methods were performed in accordance with the relevant guidelines and regulations. Using gastroscopy or postoperative pathological investigation, the diagnosis of GC was confirmed in patients. Moreover, no evidence of tumours was observed in healthy volunteers. The subjects' clinical features are presented in Supplementary Table S2.

First, 150 μl of each sample was transferred to 1 ml 96-well plates, and 600 μl of methanol was added to precipitate the protein. The mixture was then vortexed for 5 min and centrifuged at 5300 RPM for 20 min (4 °C). Second, two replicates of the 200 μl upper layer were transferred to 450 μl 96-well plates, wherein the samples were concentrated and dried via vacuum centrifugation. These two plates were used for positive and negative ion detection using untargeted metabolomics analysis. Third, the remaining upper layers of all samples were mixed and distributed into 200 μl replicates for use as quality control (QC) samples. Finally, polar metabolite analysis was performed on an Ultimate 3000 ultra-high-performance liquid chromatograph and Q-Orbitrap mass spectrometer.

### Establishment of NM score, TME score and NM-TME classifier

The differentially expressed NMRGs between GC and normal tissues were identified using the ‘limma’ packages (P < 0.05)^28^. Following this, differentially expressed genes (DEGs) were evaluated using univariate Cox regression analysis to acquire the genes with prognostic value (P < 0.05). Meanwhile, genes with prognostic significance were validated using Kaplan–Meier (KM) analysis^29^. These genes were then incorporated into a multivariate Cox regression model to calculate NM scores using the following formula: NM scores = ${\sum }_{i=1}^{n}Expi*Coefi$ (where n, Coefi and Expi denote the number of prognostic genes, the expression value and the coefficient of gene i, respectively). As for TME cells, 22 immune cell enrichment scores in patients with GC were calculated and then immune cells with prognostic significance were identified using KM analysis. Additionally, the TME score was calculated using the following formula: ${\sum }_{i=1}^{n}Expi*Coefi$ (where n, Coefi and Expi denote the number of prognostic immune cell, the infiltrating value and the coefficient of immune cell i, respectively). Gene set expression analysis (GSEA) was used to analyze the potential functions of different groups in TCGA data set. Signaling pathway differences were integrated through Kyoto Encyclopedia of Genes and Genomes (KEGG) detabase^30–32^. We then integrated NM and TME scores to construct an NM-TME classifier and classified tumours into the following subgroups: NMlow/TMEhigh, intermediate mixed (NMlow/TMElow and NMhigh/TMEhigh) and NMhigh/TMElow. The survival study using KM analysis was performed in both TCGA and GEO cohorts to determine the efficacy of the survival prediction of the signature. Furthermore, to assess the accuracy of the risk model, receiver operating characteristic (ROC) curves were generated using ‘survival ROC’ R package^33^.

### Single-cell data processing and cell–cell communication

We next analysed the relationship between NM scores and immune cells using single-cell data. GSE167297 was used to obtain single-cell transcriptome data from 10 GC samples^34^. Seurat R package was used to analyse the single-cell RNA-seq data^35^. Additionally, the ‘NormalizeData’ and ‘FindVariableFeatures’ functions in the Seurat package were used to normalise the count and expel cells containing less than 200 genes, more than 2500 genes, more than 20% of mitochondria or more than 3% of haemoglobin and then identify the 3000 highly variable genes. Moreover, the non-linear dimensional reduction was performed using the UMAP and tSNE methods. Cluster biomarkers were identified using the ‘Seurat’ package. The ‘CellChat’ R package’s method of identifying communication molecules at single-cell resolution was used to analyse the relationships between cells that were involved in communication^36^.

### Weighted gene co-expression network analysis (WGCNA)

To further investigate the potential reasons for the significant differences between the NMlow/TMEhigh and NMhigh/TMElow groups, we performed WGCNA using the R package ‘WGCNA’. A weighted value was set that conformed to the scale-free network law (scale-free R^2^ = 0.9). Topological coefficients were employed to determine the degree of dissimilarity between nodes and create a hierarchical clustering tree to separate modules. The modules with the highest correlation to the NMlow/TMEhigh and NMhigh/TMElow groups were considered key modules. Finally, functional enrichment analysis of key module genes was performed using the Metascape database^37^.

### Immune cell infiltration and immune checkpoint gene (ICG) expression between different groups

Tracking tumour immunophenotype (TIP) is a database that aids in the understanding of the mechanism of tumour immune activity and the proportion of immune cell infiltration^38^. TIP follows a seven-step ‘cancer-immunity cycle’ analysis^39^, wherein stepwise events are grouped into 23 groups with 178 stimulatory or inhibitory signature genes. Herein, TIP was used to explore the immune cell infiltration between different groups. We also examined the differential expression of common ICGs between the different groups, showing only statistically significant results.

### Tumour mutation burden (TMB) and immunotherapy response analysis

In this study, mutation data were downloaded from the TCGA database. The top 20 most frequently mutated genes in different groups were identified using the ‘maftools’ package in R. Based on the somatic mutation data in each tumour, TMB was calculated as the number of mutated bases per million bases and compared across groups. Subsequently, we also explored the survival probability between different TMB and risk scores to highlight the crucial role of TMB in GC. To further estimate the response of immunotherapy, the tumour immune dysfunction and exclusion (TIDE) algorithm was used^40^. Additionally, the Immune Checkpoint Inhibitor (ICI) Immunophenoscore (IPS) file from The Cancer Immunome Atlas Database was retrived^41^. The immunotherapeutic relevance of the signature was evaluated using IPS, a reliable tool for assessing tumour immunogenicity.

### Nomogram construction and validation

To determine if the risk score was an independent predictor of GC, univariate and multivariate Cox regression analyses were performed. Clinicopathological parameters and risk scores were considered in the development of the nomogram model for predicting the prognosis of GC using the ‘rms’ R package^42^. Additionally, we used a ROC curve to assess the validity of the established nomogram.

### Statistical analyses

The statistical analyses were conducted using strawberry-Perl and R software (R-4.13). Student t-test and Wilcoxon rank sum test was used for continuous variables and Fisher’s exact test was used for categorical variables. P < 0.05 denoted a significant outcome.

### Consent to participate and ethics approval

All patients/participants provided their written informed consent to participate in this study. The First Affiliated Hospital of Dalian Medical University’s institutional ethics committee approved this research.

## Results

### Landscape of the genetic variation of NMRGs in GC

**Figure 1:**
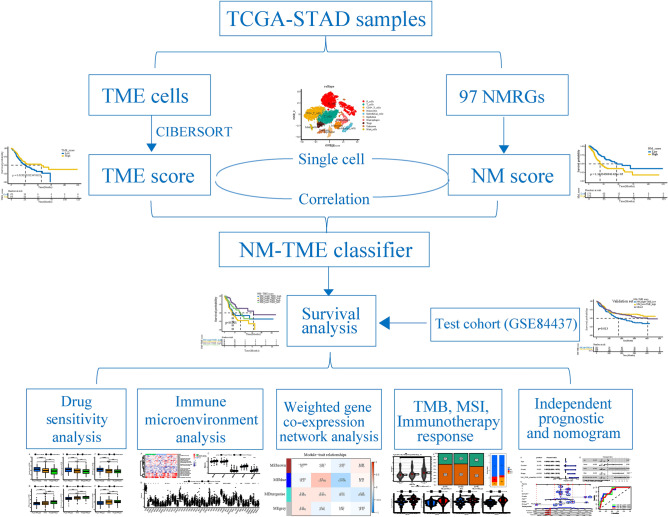
The flow chart of the study design.

Figure 1 presents the workflow of the study. Herein, 97 NMRGs were evaluated to explore their roles in GC. First, 97 NMRGs in GC were examined for copy number variations (CNVs) and somatic mutations (Supplementary Fig. 1A), with mutations identified in 161 of the 433 samples (37.18%). DPYD and XDH showed the highest mutation rate (5%) followed by CAD, AMPD3 and AK9 (4%). Furthermore, ENTPD8, ENTPD2, DNPH1, UCK1AK8 and AK1 exhibited higher frequencies of CNV amplification, whereas DCTD, IMPDH1, CDA, DPYD and AK6 exhibited higher probabilities of CNV deletions (Supplementary Fig. 1B). Supplementary Fig. 1C shows the chromosomal positions of the aforementioned CNVs. To determine the relationship between genetic variation and NMRG expression, we also compared the expression levels of 97 NMRGs between normal and tumour samples. A total of 77 genes were differentially expressed (Supplementary Fig. 1D).

To further explore the potential association between NM and GC, fresh serum samples, consisting of 33 patients with GC and 27 healthy volunteers, were collected for metabolomic analysis. Using the ‘limma’ package in R, a total of 18 differentially expressed nucleotide metabolites were identified. Among them, 1-Methyladenosine, 1-Methylguanosine, 7-Methylguanine, Allantoic acid, Cytidine, Dihydrothymine, Inosine, N2, N2-Dimethylguanosine, Pseudouridine, Uracil, Ureidopropionic acid, Uric acid and Xanthine were downregulated, whereas 5-Methylthioadenosine, 5-Methyluridine (Ribothymidine), Allantoin, N6-Methyladenosine and Uridine were upregulated in GC samples. These findings highlighted the metabolic reprogramming of NM in patients with GC (Supplementary Fig. 2).

### The prognostic values of NM and TME score

**Figure 2:**
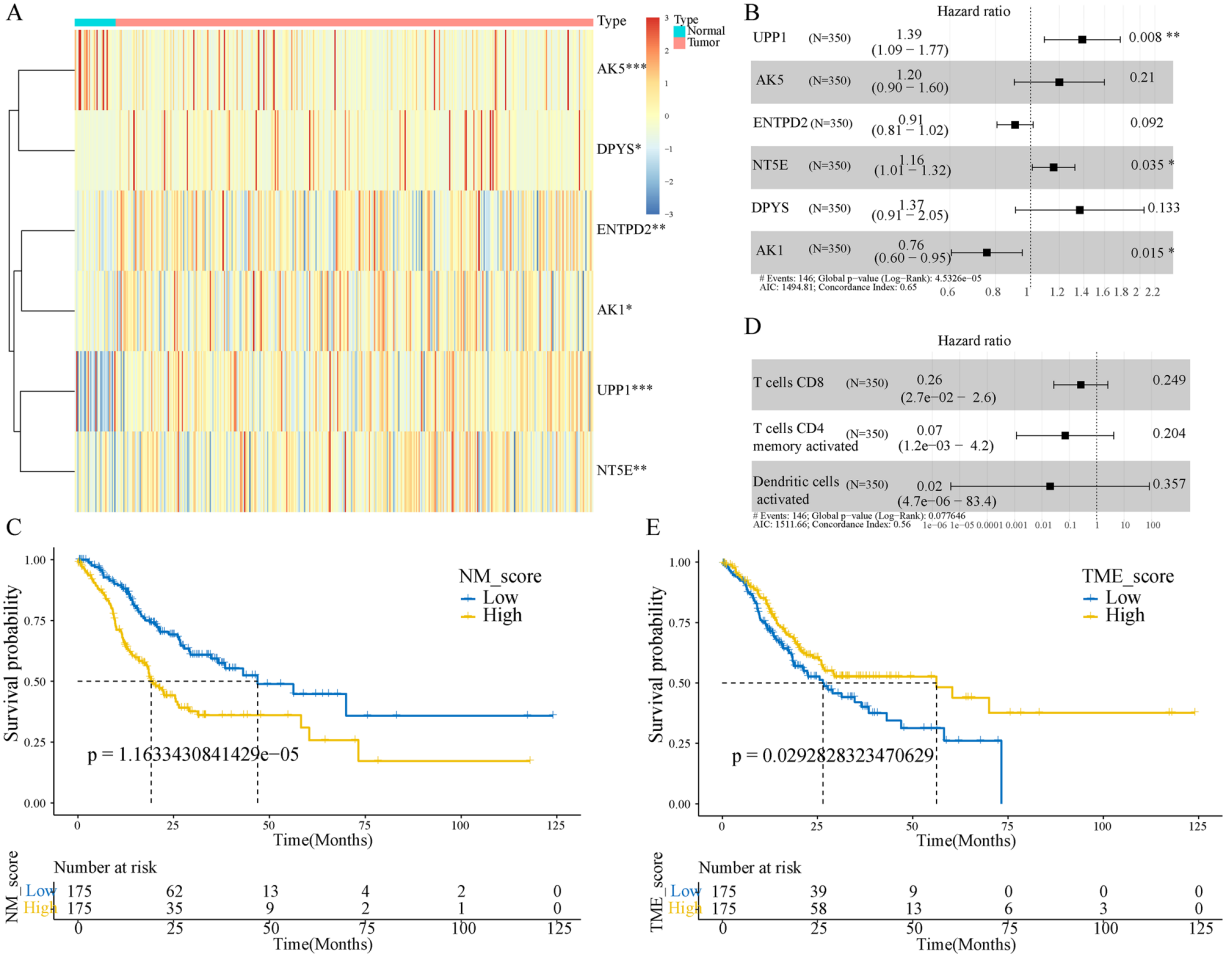
Construction of the NM- and TME-related prognostic model. (**A**) Expression levels of the six model genes. (**B**) Multivariate cox regression analysis of NM model genes. (**C**) Kaplan–Meier (KM) curves of NM-related prognostic model. (**D**) Multivariate cox regression analysis of three TME cells. (**E**) KM curves of TME-related prognostic model. *NM* nucleotide metabolism, *TME* tumour microenvironment.

To construct an NM prognostic model, we first performed a univariate Cox survival analysis on 77 differentially expressed NMRGs, of which six were statistically significant (Supplementary Table S3). Additionally, the prognostic significance of the six genes was validated using KM analysis (Supplementary Fig. 3A). Furthermore, a heatmap of the expression of the six genes in tumour and normal tissues was also drawn (Fig. 2A). We then subjected the six genes to multivariate cox analysis (Fig. 2B) and correlation coefficients were calculated to construct a model (Supplementary Table S4). The NM score was calculated for each patient, and the patients were classified into high and low score groups based on the median value. The KM curve showed that the high-risk patients had a worse prognosis (Fig. 2C). Regarding the TME prognostic model, a high infiltration of activated CD4 memory-activated T cells, CD8 T cells and activated dendritic cells (DCs) were observed to be associated with a better prognosis for patients with GC (Supplementary Fig. 3B). Similarly, these cells were subjected to multivariate cox analysis (Fig. 2D) and correlation coefficients were calculated to construct a model (Supplementary Table S5). The KM curve showed that high-TME score samples had a better survival prognosis than those with low-TME scores (Fig. 2E). GSEA revealed that the high NM score group was mainly enriched in cancer-related and classical oncogenic pathways, while the high TME score group was mainly enriched in immune-related pathways. (Supplementary Fig. 3C,D).

### Single-cell data analysis to explore the association between NM scores and TME cells

**Figure 3:**
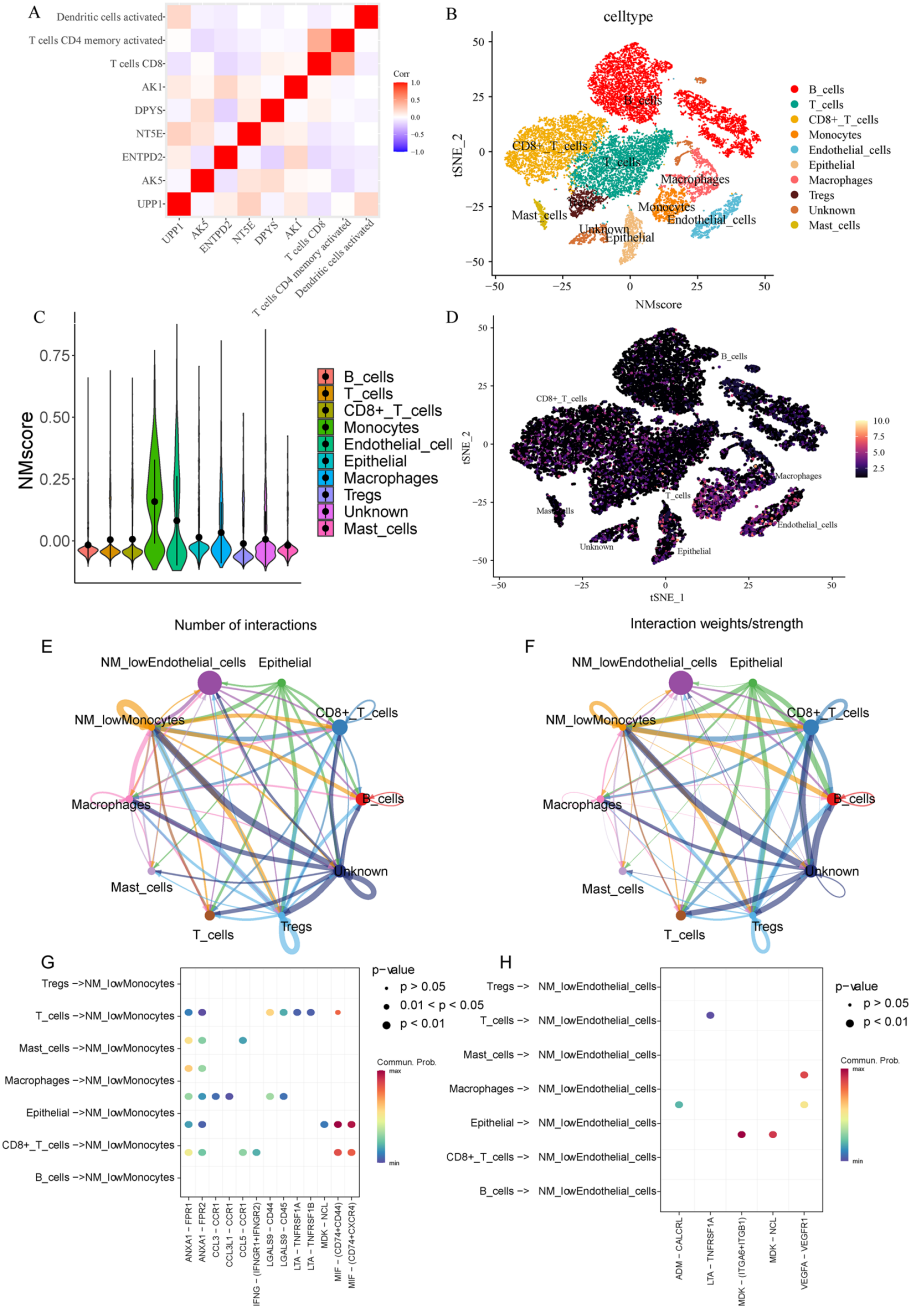
Correlation between the NM scores and TME cells. (**A**) The correlation between NMRGs and TME cells. (**B**) t-SNE plot of 10 gastric cancer samples. (**C**,**D**) Distribution of NM scores in different cell types. (**E**,**F**) The inferred signalling networks between different cell clusters. The significantly related ligand–receptor interactions of (**G**) NMlowMonocytes and (**H**) NMlowEndothelial cells. *NM* nucleotide metabolism, *TME* tumour microenvironment, *NMRGs* nucleotide metabolism-related genes.

First, we investigated the correlation between the six NM model genes and the three TME cells. We found that T cells CD8 were negatively correlated with UPP1, ENTPD2, NT5E and positively correlated with DPYS and AK1; T cells CD4 memory activated were negatively correlated with AK5, ENTPD2, NT5E, DPYS, AK1; dendritic cells were negatively correlated with AK5, ENTPD2, DPYS, and positively correlated with UPP1 (Fig. 3A). To further explore their association, we downloaded single-cell data from the GEO database, comprising 10 GC samples. The clustering and annotated results are presented in Fig. 3B. Subsequently, we calculated the NM scores in different cell types and found that the NM scores were significantly higher in monocytes and endothelial cells than in B cells, T cells, CD8+ T cells, epithelial, macrophages, Tregs and mast cells (Fig. 3C,D). Based on the NM score, monocytes and endothelial cells were divided into low NM score, medium NM score and high NM score monocytes and endothelial cells for cell communication analysis. The monocytes and endothelial cells with low NM scores had more abundant communication with other immune cells (Fig. 3E–H). Therefore, low NM scores could have a synergistic effect with high TME scores and combining the NM model with the TME model may be a feasible method.

### NM-TME classifier construction and validation

**Figure 4:**
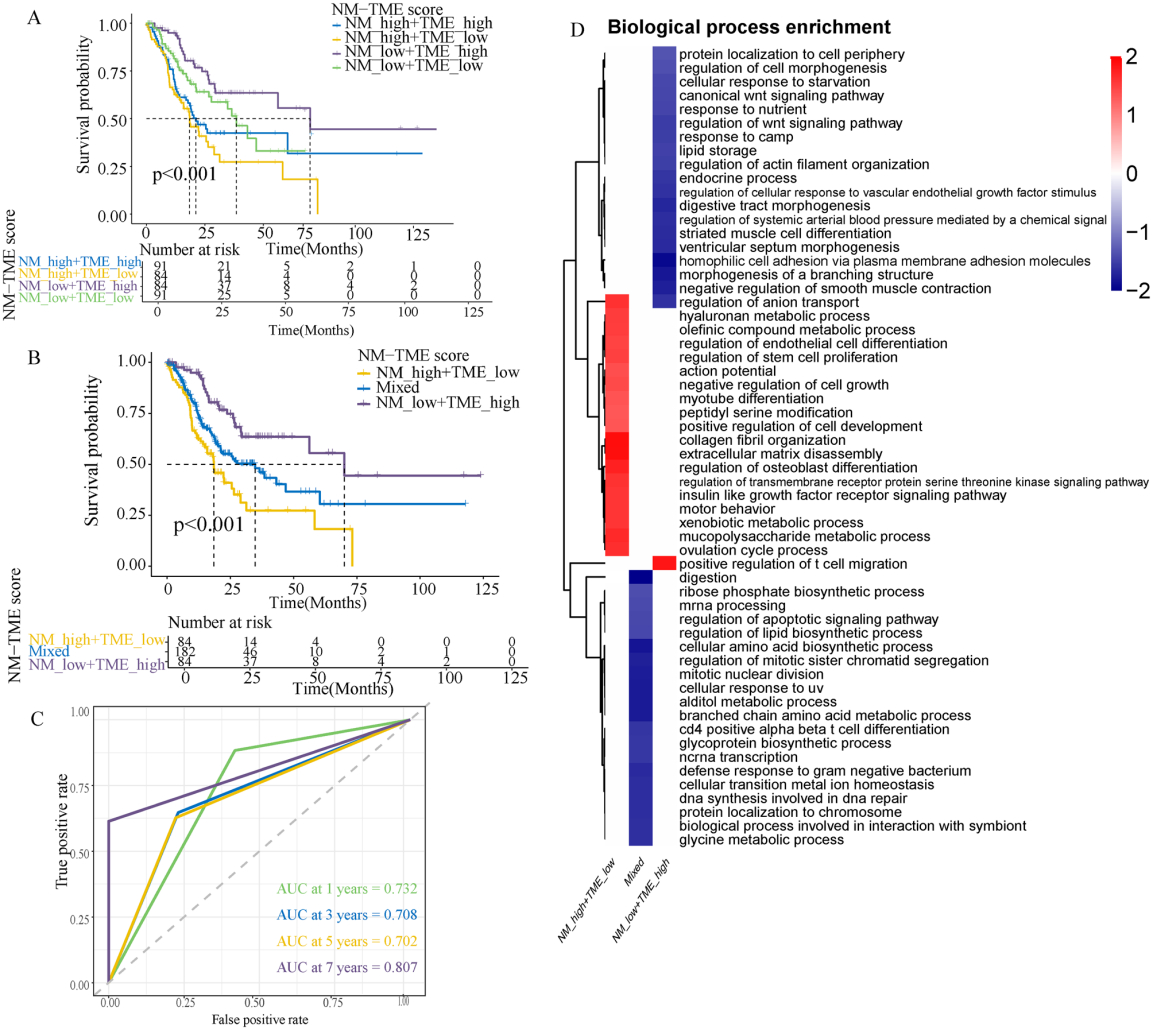
Construction of the NM-TME classifier and functional enrichment analysis. (**A**) Survival analysis of the four subgroups was obtained based on the NM-TME classifier. (**B**) Survival analysis after merging the NMlow/TMElow and NMhigh/TMEhigh subgroups. (**C**) Receiver operating characteristic (ROC) curve of the NM-TME classifier. (**D**) Functional enrichment analysis of the three subgroups was obtained based on the NM-TME classifier. *NM* nucleotide metabolism, *TME* tumour microenvironment.

Next, we constructed the NM-TME classifier by combining the NM and TME scores. It divided patients with GC into four categories: NMhigh/TMEhigh, NMhigh/TMElow, NMlow/TMEhigh and NMlow/TMElow. Survival analysis revealed that the NMhigh/TMElow group had a poorer prognosis while the NMlow/TMEhigh group had a better prognosis among the groups (Fig. 4A). Patients in the NMhigh/TME high and NMlow/TME low subgroups showed less divergent prognoses. As a result, we combined them to form a mixed subgroup (Fig. 4B). Additionally, the area under the curve (AUC) values of the NM-TME classifier were 0.732, 0.708, 0.702 and 0.807 for 1, 3, 5 and 7 years, respectively (Fig. 4C), indicating that the NM-TME classifier plays a significant role in the survival prediction of patients with GC.

Furthermore, we also verified the prognostic significance of the NM-TME classifier in the GEO cohort, which revealed significant prognostic differences between the groups (Supplementary Fig. 4A). Moreover, the evaluation of the predictive performance of the classifier under different clinical features in the TCGA cohort revealed good predictive performance (Supplementary Fig. 4B).

### Functional enrichment analysis and WGCNA

Functional enrichment of the three groups revealed that the NMhigh/TMElow group was mainly enriched in the regulation of the olefinic compound metabolic process, endothelial cell differentiation and stem cell proliferation, while the NMlow/TMEhigh was majorly positively associated with the positive regulation of T cell migration and negatively associated with the canonical Wnt signalling pathway (Fig. 4D).

**Figure 5:**
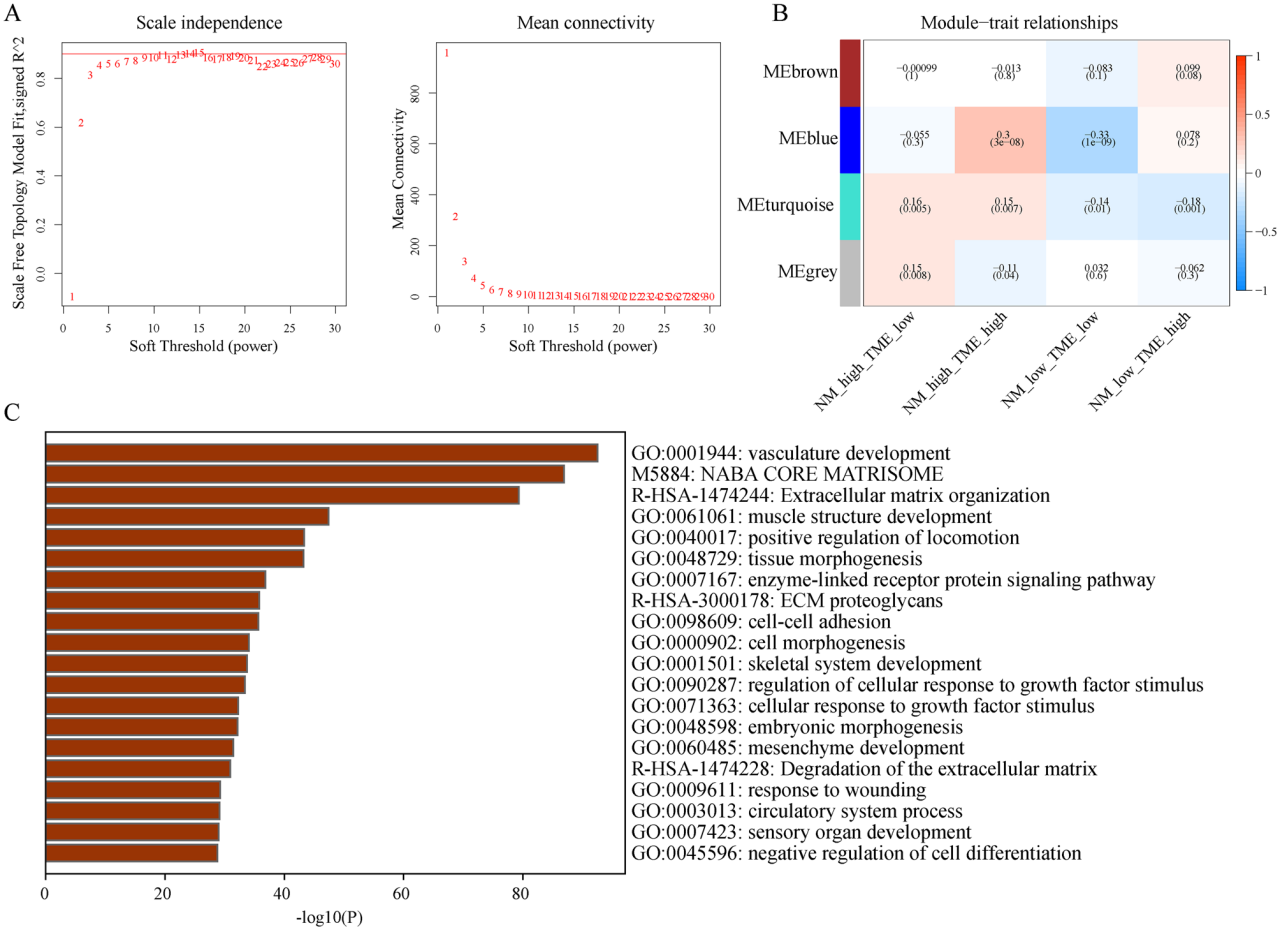
Exploring key module eigengenes associated with the NMlow/TMEhigh and NMlow/TMElow groups using weighted gene co-expression network analysis. (**A**) Evaluation of the scale-free fit index for differing soft-thresholding powers (β) and examination of the connectivity of various soft-thresholding powers. (**B**) A heatmap depicts the association between module eigengenes and various subgroups. (**C**) Functional enrichment analysis of key module eigengenes. *NM* nucleotide metabolism, *TME* tumour microenvironment.

Furthermore, WGCNA identified four modules (Fig. 5A,B). Among them, the turquoise module was most relevant and opposite to each other for the NMlow/TMEhigh and NMhigh/TMElow groups. Therefore, the turquoise module gene could be associated with significantly different prognoses between the NMlow/TMEhigh and NMhigh/TMElow groups. Using the Metascape database, enrichment analysis of these genes revealed that they were mainly enriched in vasculature development, NABA core matrisome and extracellular matrix organization (Fig. 5C).

### Differences in immune cell infiltration and ICG expression based on the NM-TME classifier

**Figure 6:**
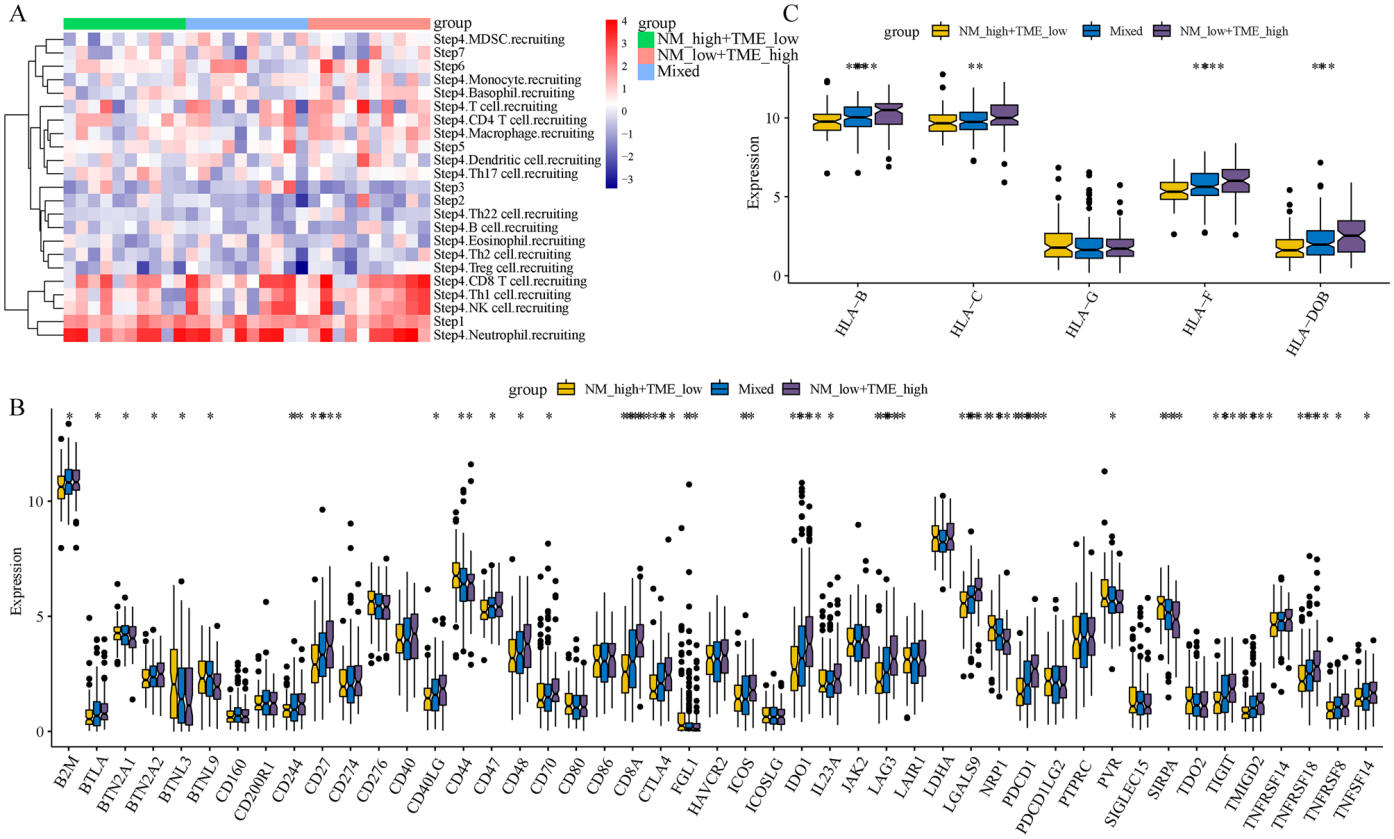
Immune status of different subgroups based on the NM-TME classifier. (**A**) Differences in immune cell infiltration. (**B**) Differences in ICGs. (**C**) Differences in antigen presentation-related genes in different subgroups. *NM* nucleotide metabolism, *TME* tumour microenvironment, *ICG* immune checkpoint gene.

First, we compared the abundance of immune cell infiltration between the different groups. The immune cell infiltration was more abundant in the NMlow/TMEhigh group, especially CD8 T cells, Th1 cells, NK cells, CD4 T cells and macrophages (Fig. 6A). Notably, the better prognosis in the NMlow/TMEhigh group could be attributed to the abundant immune cell infiltration. Meanwhile, we also explored whether the expression of common ICGs differed between the groups. Most ICGs were differentially expressed between the groups, with high expression observed in the NMlow/TMEhigh group (Fig. 6B). These differentially expressed ICGs could be potential therapeutic targets. Additionally, it also suggests that NMlow/TMEhigh patients may benefit more from immune checkpoint blockade (ICB) therapy. HLA is a polygenic and polymorphic complex involved in antigen presentation^43^. Figure 6C shows that HLA-B, HLA-C, HLA-F and HLA-DOB were expressed the highest in the NMlow/TMEhigh group.

### Intergroup differences in cancer somatic mutations

**Figure 7:**
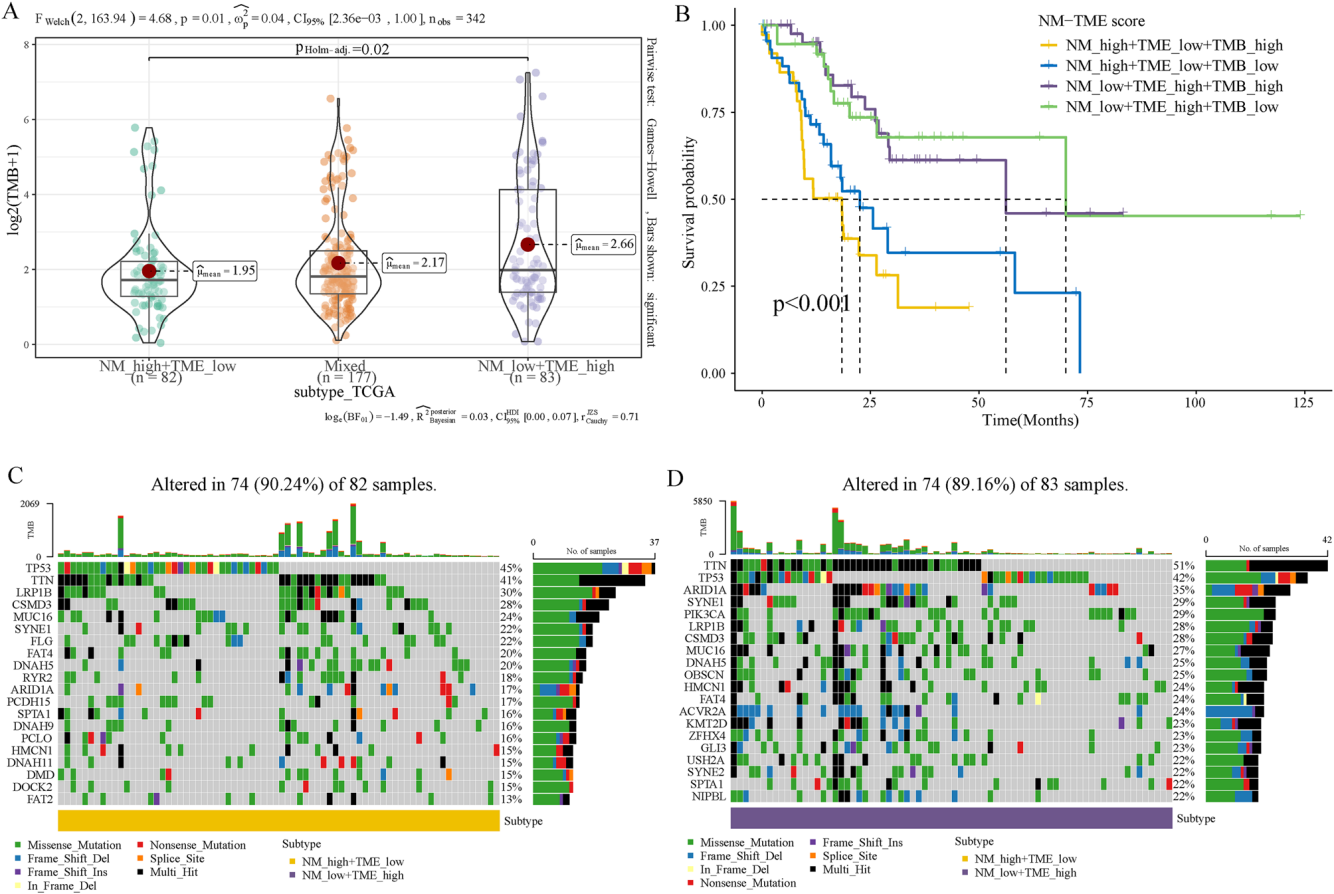
TMB analysis. (**A**) Comparison of TMB among the defined subgroups. (**B**) Survival analysis based on the NM-TME classifier and TMB. The top 20 mutation genes of the (**C**) NMhigh/TMElow and (**D**) NMlow/TMEhigh groups. *NM* nucleotide metabolism, *TME* tumour microenvironment, *TMB* tumour mutation burden.

Numerous studies have demonstrated the association between somatic mutations in tumour genomes and the response to immunotherapy^44^. We therefore examined the TMB distributions among the various groups based on the NM-TME classifier. The NMlow/TMEhigh group had a higher TMB, while the NMhigh/TMElow group had a lower TMB, indicating that the NMlow/TMEhigh group may benefit more from immunotherapy (Fig. 7A). Additionally, the NMhigh/TMElow/TMBhigh group had a lower prognosis than patients in the other groups (Fig. 7B). Figure 7C,D display the top 20 genes with high mutation frequencies in the NMlow/TMEhigh and NMhigh/TMElow groups.

### Personalised treatment based on the NM-TME classifier

**Figure 8:**
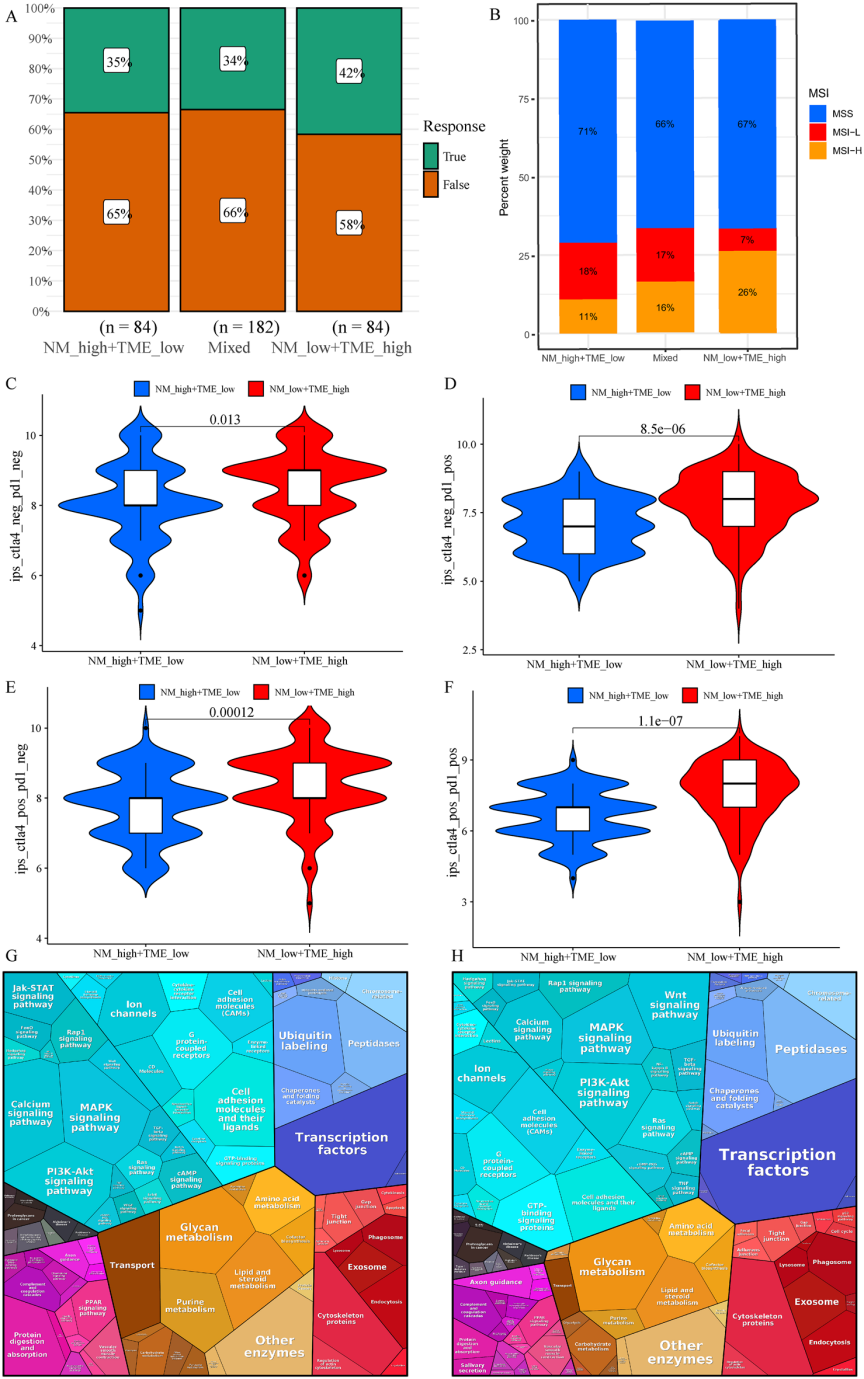
The role of NM-TME classifier in immunotherapy. (**A**) Proportion of response to immunotherapy in different groups. (**B**) Proportion of MSI in different groups. (**C**–**F**) Comparison of the relative distribution of IPS across groups with high NM/low TME and low NM/high TME. Functional analysis in the NMlow/TMEhigh group (**G**) and responder of patients under immunotherapy (**H**) illustrated using Proteomaps. A little polygon represents a unique KEGG pathway. *NM* nucleotide metabolism, *TME* tumour microenvironment, *MSI* microsatellite instability, *IPS* immunophenoscore.

Considering that drugs targeting PD-1 and CTLA-4 have recently received approval for the treatment of several cancers, we evaluated whether the NM-TME classifier could predict patients’ reactions to immunotherapy. The patients in the NMlow/TMEhigh group were observed to have a better response rate to immunotherapy than the other two groups (Fig. 8A). Microsatellite instability-high (MSI-H) is a potential predictor of immunotherapy response targeting PD-1 or its ligand PD-L1^45^. Accordingly, the proportion of MSI-H in the NMlow/TMEhigh group was higher than that in the other two groups (Fig. 8B). Additionally, we investigated the relationship between the NM-TME classifier and IPS in patients with GC to predict the response to ICIs. Figure 8C–F presents the differences in the results of CTLA-4/PD-1 inhibitor treatment between the NMlow/TMEhigh and Nmhigh/TMElow groups. The NMlow/TMEhigh group has higher IPS scores, implying more immunogenicity in the NMlow/TMEhigh group. Furthermore, we performed a difference analysis between the immunotherapy-responsive and non-responsive groups and also the NMlow/TMEhigh and NMhigh/TMElow groups. DEGs were then analysed using the Proteomaps 2.0 database^46^. Notably, the pattern of proteomap in the NMlow/TMEhigh group and immunotherapy-responsive groups were similar (Fig. 8G,H). These findings suggest that the NM-TME classifier can be used to predict patients’ responses to immunotherapy.

Given that targeted therapy is an effective approach in the treatment of GC, it has important clinical applications and prospects. We, therefore, investigated whether the NM-TME classifier could predict drug sensitivity in patients with GC. The NMhigh/TMElow group benefited more from Imatinib, Midostaurin and OSI-906 (Linsitinib), while those in the NMlow/TMEhigh group benefited more from Paclitaxel, Methotrexate and Camptothecin (Supplementary Fig. 5A–F).

### Nomogram development and verification

**Figure 9:**
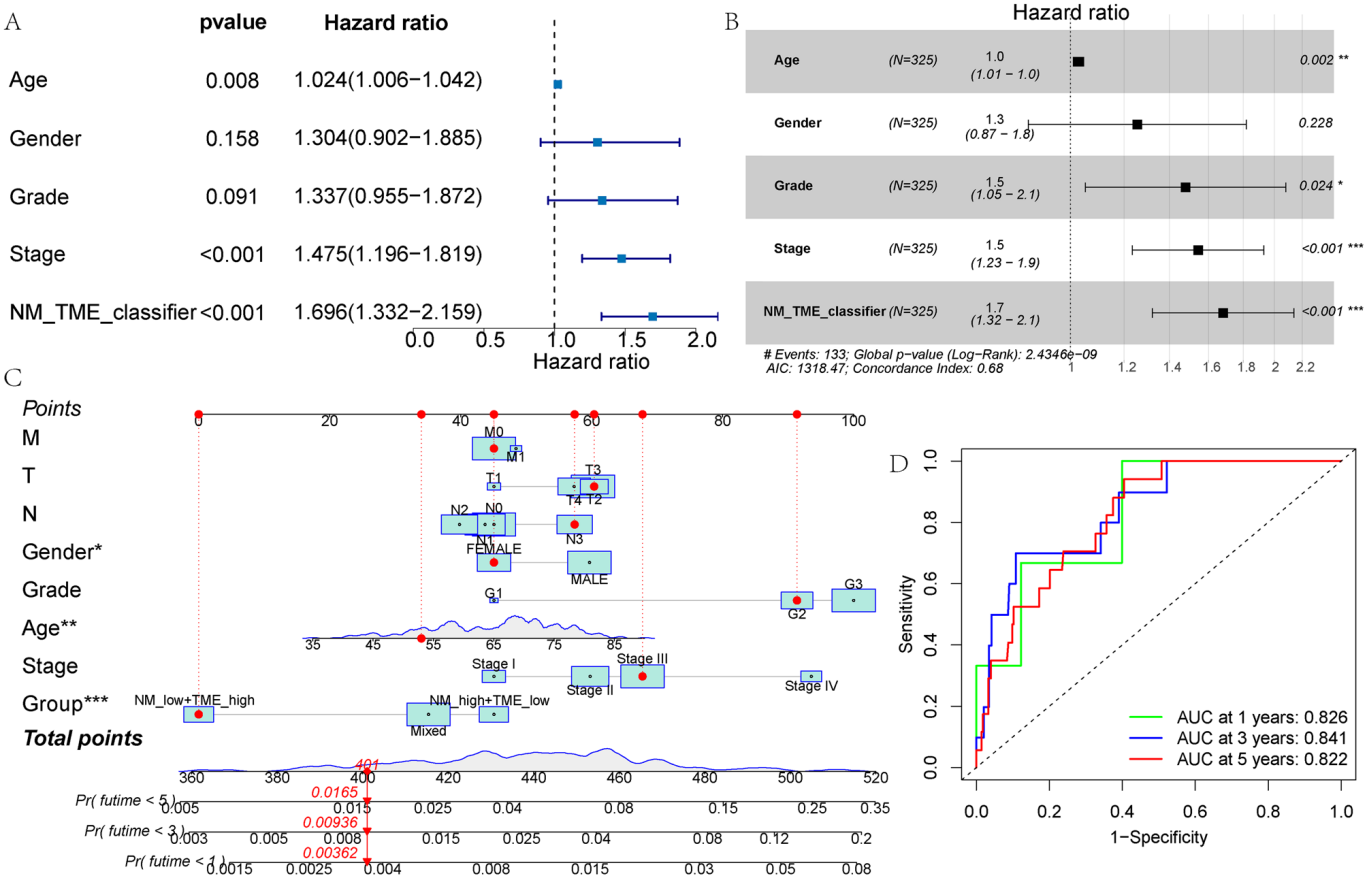
Construction of a nomogram. (**A**,**B**) Forest map of univariable and multivariable Cox regression in the test cohort. (**C**) Nomogram based on the NM-TME classifiers and clinical features. (**D**) Receiver operating characteristic (ROC) curves of the nomogram model in predicting the 1–5 years survival rate. *NM* nucleotide metabolism, *TME* tumour microenvironment.

Univariate and multivariate Cox regression analyses indicated that the NM-TME classifier was an independent predictor of prognosis with the highest hazard ratio (HR) (Fig. 9A,B). Following this, the NM-TME classifier and clinical features were combined to construct a nomogram. To predict the survival of patients with GC over 1 to 5 years, the values of each variable can be added to obtain the total score (Fig. 9C). Moreover, the AUC values of the nomogram for 1-, 3- and 5-year OS were 0.826, 0.841 and 0.822, respectively (Fig. 9D).

## Discussion

Owing to its high morbidity, the poor incidence of early diagnosis and low survival rate, GC poses a severe threat to the populations worldwide^47^. Moreover, the development of tumours is consistent with abnormal metabolism^48^. Recent studies have demonstrated that aberrant NM speeds up the progression of tumours while suppressing the TME’s normal immune response^49,50^. Therefore, to treat malignancies and prevent recurrence and metastasis, the intervention or regulation of molecular pathways related to aberrant NM in malignant cells has emerged as a novel therapeutic strategy^20^. The TME has a vital role in tumour development, growth, metastasis and therapeutic response^51^. As a therapeutic target in tumours, TME has attracted significant research and clinical interest^52^. However, very few studies have reported on the use of NM-TME characteristics in predicting GC prognosis and treatment response. In this study, we combined the NM and TME features, for the first time, to construct the NM-TME classifier, which consists of three types of immune cells and six NMRGs. Currently, in various malignancies, next-generation sequencing is becoming a complementary diagnostic tool that guides decision-making to achieve precise and personalised therapy regimens. Accordingly, based on our constructed NM-TME signature, clinicians can quantify specific immune cells and target genes using sequencing technology and related algorithms to effectively predict prognosis, immunotherapy response and targeted therapy response in patients with GC.

First, we constructed an NM prognostic model with six genes, namely *AK1, DPYS, NT5E, ENTPD2, AK5* and *UPP1*. Second, we constructed a prognostic model of the TME, consisting of activated DCs, activated CD4 memory T cells and CD8 T cells. Both prognostic models classified patients with GC into two groups with significant prognostic differences. Additionally, patients with high NM scores had a poor prognosis and majorly played a role in cancer-related pathways. In contrast, patients with high TME scores had a better prognosis and were mainly involved in immune-related pathways. Therefore, we speculated that these two models could have synergistic effects.

Moreover, a correlation was observed between the six NMRGs and three TME cells that were involved in the construction of the model. Additionally, we used single-cell data to further explore the association between NM scores and immune cells. After QC, clustering, and annotation of the single cell data, we calculated the NM scores in each cell type. The results showed that the NM scores in monocytes and endothelial cells were significantly higher than in other cells. We then divided monocytes and endothelial cells into monocytes and endothelial cells with high NM, medium NM and low NM scores. Furthermore, cell communication analysis also showed that low NM score monocytes and endothelial cells were more closely related to other immune cell types. Thus, these findings suggest a strong association between low NM scores and high TME scores, highlighting their synergistic effect on the prognosis of patients with GC.

Based on the above analysis, we constructed an NM-TME classifier that can classify patients with GC into different subgroups based on NM and TME scores. Survival analysis showed significant differences in prognosis between the groups, which was consistent with the results of the test set. A key module was identified to be significantly associated with the NMlow/TMEhigh and NMhigh/TMElow groups via WGCNA, which could be responsible for their significant differences. Moreover, the key module genes were mainly enriched in vasculature development, NABA core matrisome and extracellular matrix organization.

We then examined the immune status of the different groups of patients based on the NM-TME classifier. Patients in the NMlow/TMEhigh group had a higher abundance of immune cell infiltration, which may be the reason for better prognosis in NMlow/TMEhigh group. Moreover, ICB therapy as emerging immunotherapy target has demonstrated therapeutic efficacy in the treatment of human malignancies^53^. Herein, most ICGs were highly expressed in the NMlow/TMEhigh group. These differentially expressed ICGs could be potential therapeutic targets, suggesting that patients in the NMlow/TMEhigh group may benefit more from ICB.

TMB has been demonstrated to be utilised as a predictor of ICB efficacy and has become a biomarker in certain types of cancer to identify patients who might benefit from immunotherapy^44,54,55^. On analysing TMB values, the NMlow/TMEhigh group exhibited a higher TMB, while a converse trend was observed in the NMhigh/TMElow group. Thus, patients in the NMlow/TMEhigh group can be considered more sensitive to ICB treatment.

According to recent studies, blocking PD-1 is not inferior to chemotherapy^56^ and combining a PD-1 inhibitor with chemotherapy improves survival in individuals with advanced GC compared to chemotherapy alone^57^. Nevertheless, anti-PD-1 immunotherapy has been reported to be efficient in 15–60% of patients. Therefore, we investigated the relationship between the NM-TME classifier and the outcome of CTLA-4/PD-1 inhibitor therapy. The NMhigh/TMElow group had a better response to CTLA-4/PD-1 inhibitor therapy. Furthermore, validation using the TIDE database revealed that the immunotherapy response rate and proportion of MSI-H were higher in the NMlow/TMEhigh group. Meanwhile, the pattern of proteomap in the NMlow/TMEhigh group and immunotherapy responder were also similar. These results further demonstrate that patients in the NMlow/TMEhigh group are more sensitive to immunotherapy and that the NM-TME classifier can effectively predict patients’ response to immunotherapy.

In addition, the NM-TME classifier was able to predict chemotherapy drug sensitivity. The NMhigh/TMElow group benefited more from Imatinib, Midostaurin and OSI-906 (Linsitinib), while patients in the NMlow/TMEhigh group benefited more from Paclitaxel, Methotrexate and Camptothecin. Drug resistance is a major challenge in cancer treatment. It is speculated that drug resistance in cancer is driven by genetic mutations. Despite the unclear mechanism of drug resistance, there is evidence for an important role of reversible proteomic and epigenetic mechanisms in drug resistance. Additionally, mechanisms mediated by the TME and tumour heterogeneity greatly contribute to cancer therapy resistance^58^. Tyrosine kinase inhibitors (TKIs) therapy, such as Imatinib and OSI-906 (Linsitinib), play a role in the TME remodelling and enhance therapeutic response, but TME changes can also induce drug resistance and promote tumour growth. The higher resistance to Imatinib and OSI-906 (Linsitinib) in patients in the NMlow/TMEhigh group could be attributed to their more abundant immunosuppressive TMEs, such as macrophages, neutrophils, Tregs and myeloid-derived suppressor cells (MDSCs)^59^. It has been reported that Midostaurin may enhance anti-tumour effects by modulating the distribution of immune cells in the TME. Thus, resistance to Midostaurin could also be associated with the anti-tumour immunity of neutrophils and MDSCs^60^. Paclitaxel can promote the polarisation to DCs and the proliferation and activity of CD8+ T cells and NK cells to exert stronger anti-tumour effects, which leads to a higher sensitivity to paclitaxel in the NMlow/TMEhigh group^61^. Methotrexate is an anti-tumour agent that interferes with folic acid metabolism. Studies have reported that higher NADPH levels in acute myeloid leukaemia promote Methotrexate resistance and that NADPH is involved in nucleotide synthesis^62^, which could be associated with greater sensitivity in the NMlow/TMEhigh group. However, the corresponding mechanisms involved in GC require further investigation.

The multifactorial analysis demonstrated that the NM-TME classifier is an independent prognostic factor for patients with GC, with excellent prognostic predictive power. Finally, to fully exploit the prognostic potential of the NM-TME classifier, the survival rate of patients with GC was quantified after constructing a nomogram based on the signature and clinical features. The ROC curve illustrated the high-precision predictive capability of the nomogram.

To our knowledge, this is the first study to use bioinformatics to combine TME and NM features to analyse their role in GC prognosis, immunotherapy and chemotherapy. Nonetheless, this investigation is not without its drawbacks. The data used herein are from online databases, namely TCGA and GEO. Thus, these findings require further validation using real prospective clinical cohorts. Furthermore, basic investigations on the function of the TME and NM in the aetiology and progression of GC are required as the current understanding of this topic is limited.

## Conclusion

In our study, an NM-TME signature was constructed by combining NM and TME features to predict the prognosis, immunotherapy and chemotherapy effects of patients with GC. This classifier has been well-validated from different points of view.

## Supplementary Information

Supplementary Information.

## Data Availability

The datasets analysed in this study are publicly available from the TCGA and GEO (GSE84437, https://www.ncbi.nlm.nih.gov/geo/query/acc.cgi?acc=GSE84437) databases. Furthermore, the raw data and analytic technologies used in this study can be obtained from the corresponding author and first author upon reasonable request.
