# Supplementary material for: The combined signatures of the tumour microenvironment and nucleotide metabolism-related genes provide a prognostic and therapeutic biomarker for gastric cancer
Source: Sci Rep. 2023 Apr 24;13:6622. doi: 10.1038/s41598-023-33213-z (PMC10126105; doi:10.1038/s41598-023-33213-z)
Supplement: Supplementary file 1 — Supplementary Information. [file 41598_2023_33213_MOESM1_ESM.pdf]

The combined signatures of the tumour microenvironment and nucleotide metabolism-related genes provide a prognostic and therapeutic biomarker for gastric cancer

Jifeng Liu[1]†, Lei Zhong[1]†, Dawei Deng[2]†, Yunshu Zhang[3]*, Qihang Yuan[1]*, Dong Shang[1]*

[1]Department of General Surgery, The First Affiliated Hospital of Dalian Medical University, Dalian, Liaoning, China

[2]Department of Hepato-Biliary-Pancreas, Affiliated Hospital of North Sichuan Medical College, Nanchong, China

[3]Department of Traditional Medicine, The First Affiliated Hospital of Dalian Medical University, Dalian, Liaoning, China

†These authors have contributed equally to this work and share first authorship

***Correspondence:**

Prof. Dong Shang, E-mail: shangdong@dmu.edu.cn;

Dr. Qihang Yuan, E-mail: qihangdy@163.com;

Dr. Yunshu Zhang, E-mail: zys1986062186@163.com.

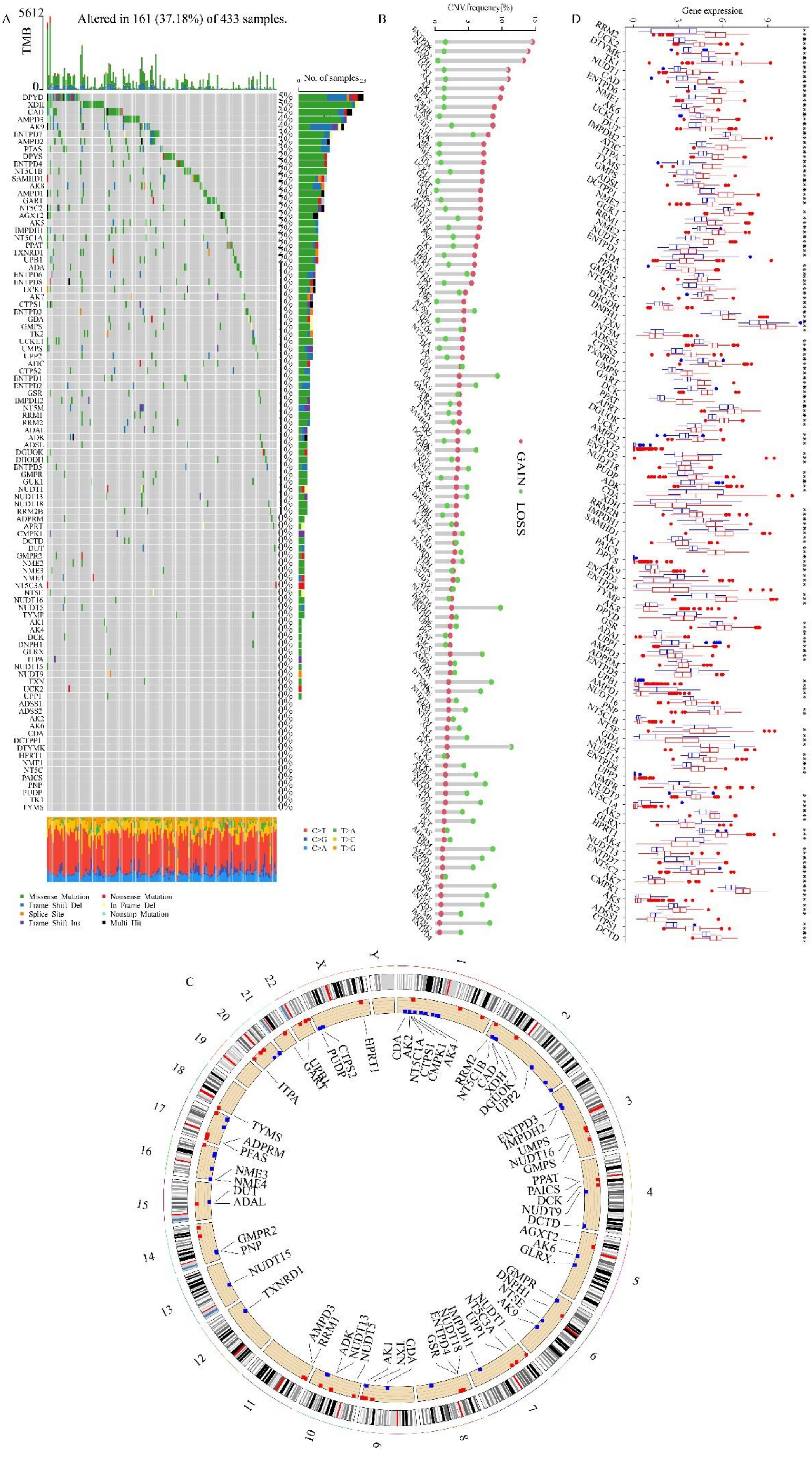
A
Altered in 161 (37.18%) of 433 samples.
TMB
No. of samples
Missense Mutation
Nonsense Mutation
Frame Shift Del
In Frame Del
Splice Site
Nonstop Mutation
Frame Shift Ins
Multi Hit
B
CNV.frequency(%)
GAIN
LOSS
C
D
Gene expression
Type
Normal
Tumor

Supplementary Figure 1. Genetic variation of NMRG in GC. (A) 161 of 433 patients have genetic alterations of NMRG. (B) CNV mutation frequency of NMRGs. (C) Position of CNV change of NMRG on chromosome. (D) Difference in NMRG expression between normal and GC.

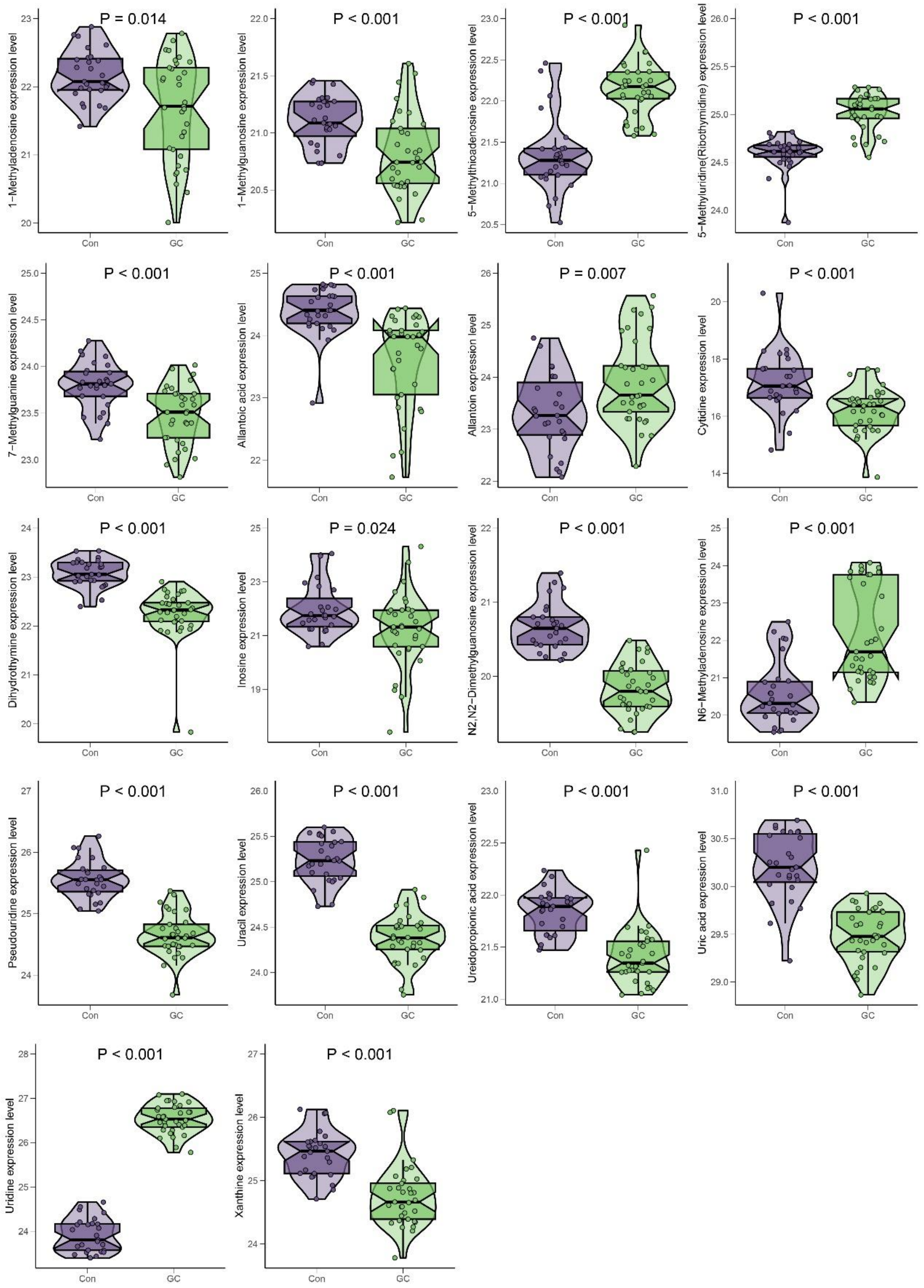

Supplementary Figure 2. 18 differentially expressed nucleotide metabolites (1-Methyladenosine, 1-Methylguanosine, 5-Methylthioadenosine, 5-Methyluridine (Ribothymidine), 7-Methylguanine, Allantoic acid, Allantoin, Cytidine,

Dihydrothymine, Inosine, N2, N2-Dimethylguanosine, N6-Methyladenosine, Pseudouridine, Uracil, Ureidopropionic acid, Uric acid, Uridine, Xanthine).

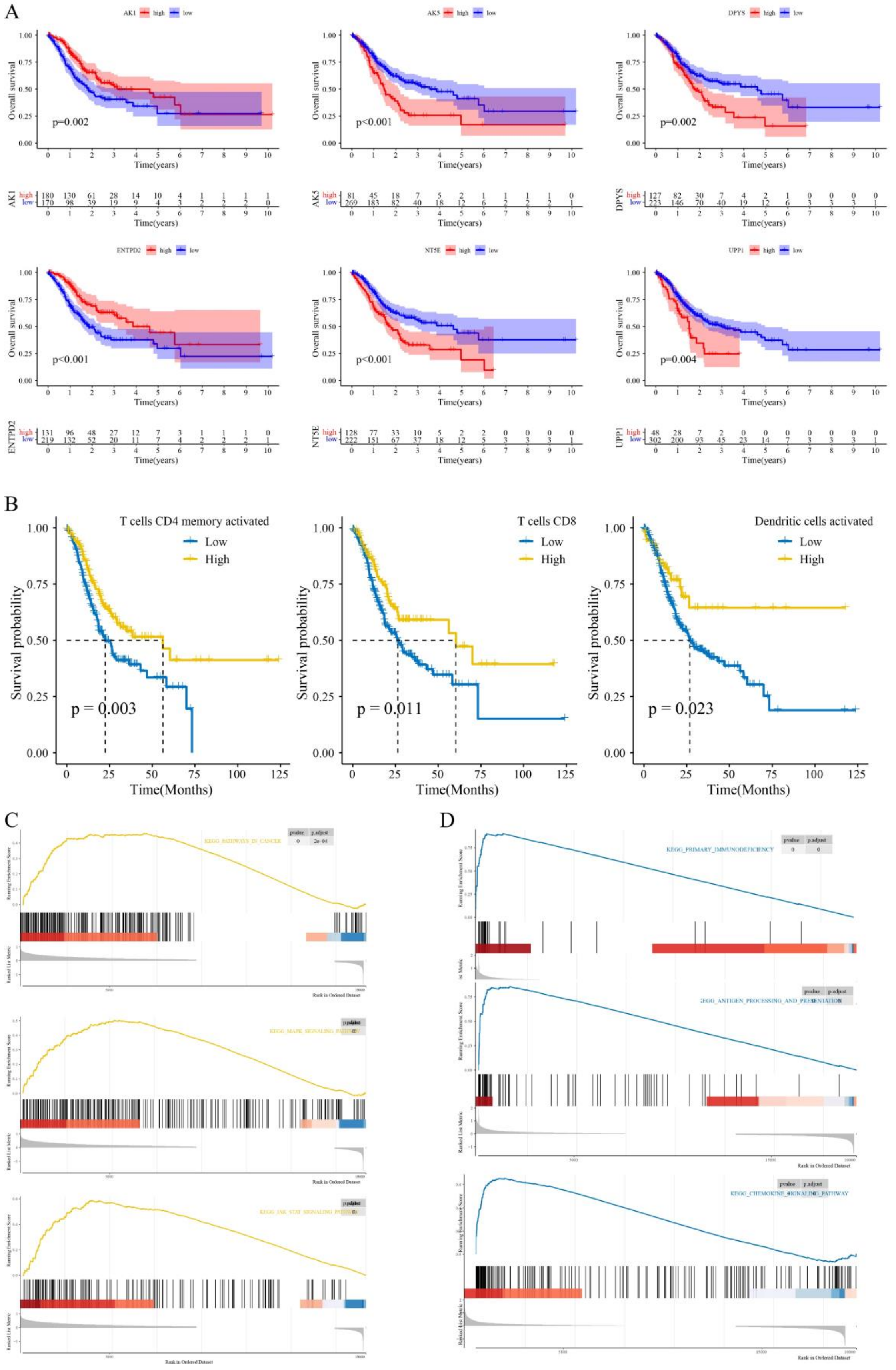

Supplementary Figure 3. (A) KM analysis of 6 NMRGs. (B) KM analysis of 3 immune cells. (C-D) GSEA analysis

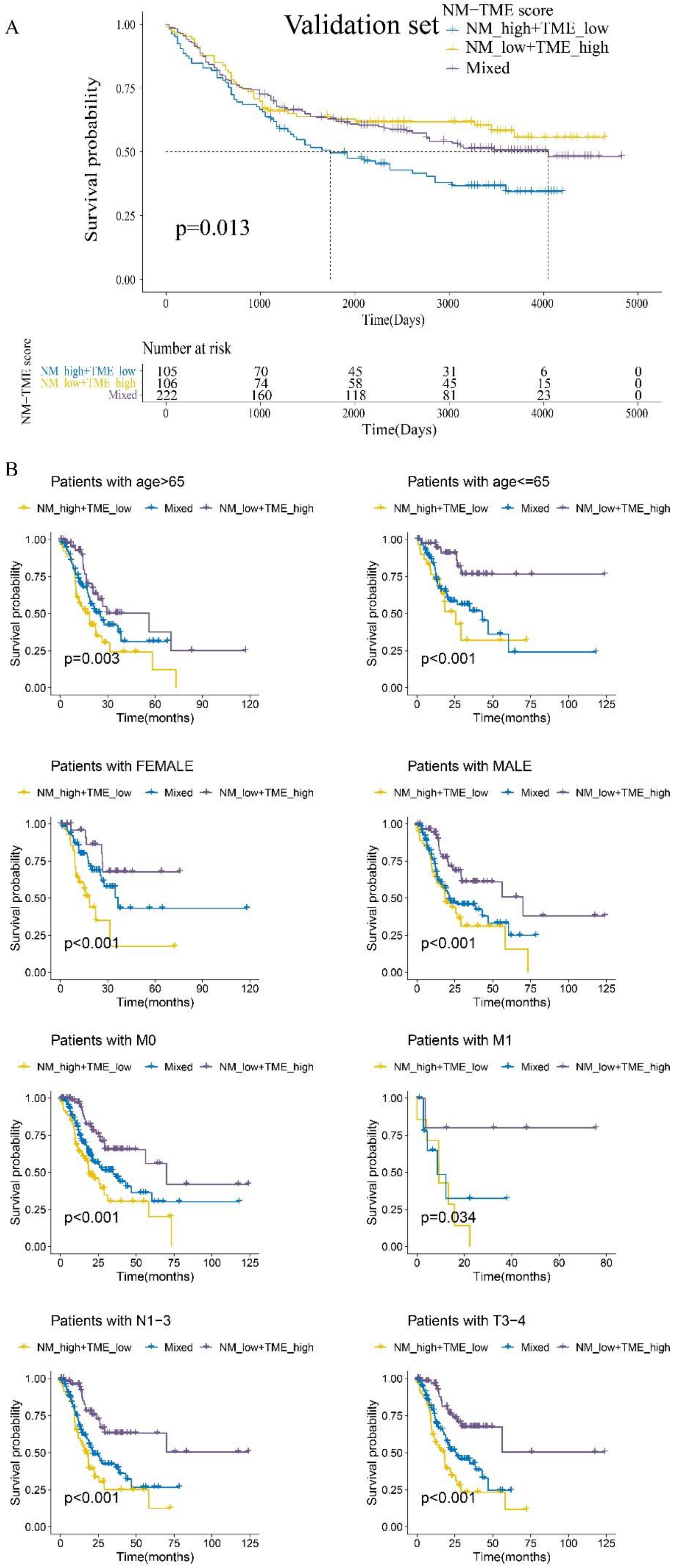
A
Validation set
NM−TME score
NM_high+TME_low
NM_low+TME_high
Mixed
Survival probability
p=0.013
Time(Days)
Number at risk
NM_high+TME_low 105 70 45 31 6 0
NM_low+TME_high 106 74 58 45 15 0
Mixed 222 160 118 81 23 0
B
Patients with age>65
p=0.003
Patients with age<=65
p<0.001
Patients with FEMALE
p<0.001
Patients with MALE
p<0.001
Patients with M0
p<0.001
Patients with M1
p=0.034
Patients with N1−3
p<0.001
Patients with T3−4
p<0.001
NM_high+TME_low
Mixed
NM_low+TME_high
Time(months)

Supplementary Figure 4. (A) Survival analysis in GEO cohort. (B) Survival analysis of patients with different clinical characteristics.

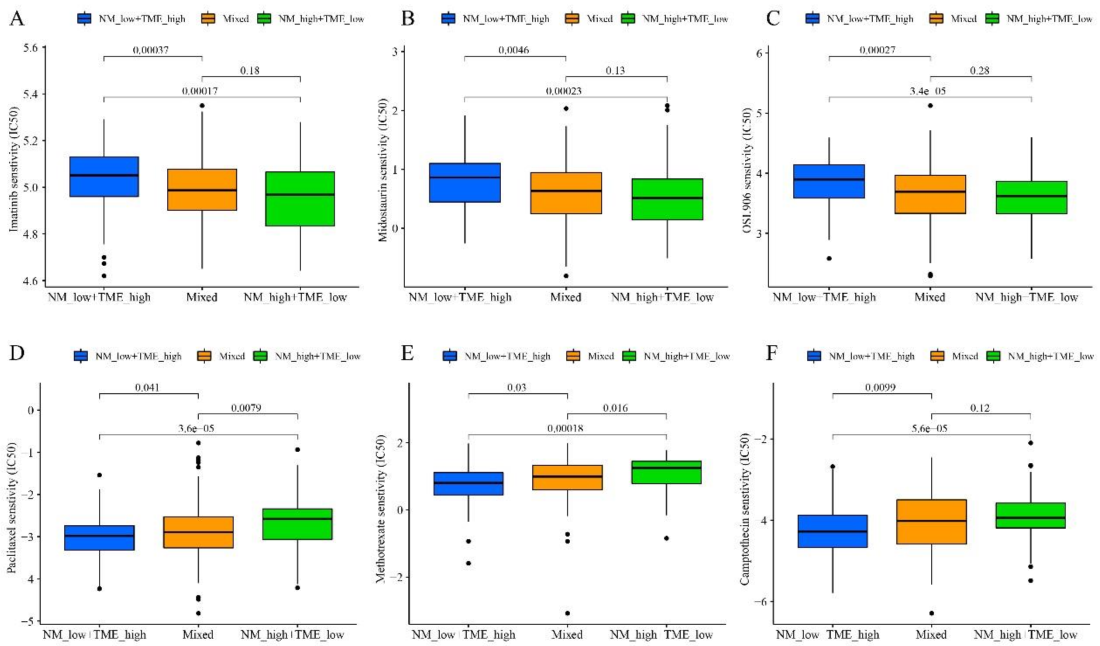

Supplementary Figure 5. Drug sensitivity analysis.

REACTOME_METABOLISM_OF_NUCLEOTIDES
ADA
ADAL
ADK
ADPRM
ADSL
ADSS1
ADSS2
AGXT2
AK1
AK2
AK4
AK5
AK6
AK7
AK8
AK9
AMPD1

AMPD2
AMPD3
APRT
ATIC
CAD
CDA
CMPK1
CTPS1
CTPS2
DCK
DCTD
DCTPP1
DGUOK
DHODH
DNPH1
DPYD
DPYS
DTYMK
DUT
ENTPD1
ENTPD2
ENTPD3
ENTPD4
ENTPD5
ENTPD6
ENTPD7
ENTPD8
GART
GDA
GLRX
GMPR
GMPR2
GMPS
GSR
GUK1
HPRT1
IMPDH1
IMPDH2
ITPA
NME1
NME2
NME3
NME4
NT5C

NT5C1A
NT5C1B
NT5C2
NT5C3A
NT5E
NT5M
NUDT1
NUDT13
NUDT15
NUDT16
NUDT18
NUDT5
NUDT9
PAICS
PFAS
PNP
PPAT
PUDP
RRM1
RRM2
RRM2B
SAMHD1
TK1
TK2
TXN
TXNRD1
TYMP
TYMS
UCK1
UCK2
UCKL1
UMPS
UPB1
UPP1
UPP2
XDH

Supplementary Table 1. Gene list of 97 NMRGs.

	GC (n=33)	Healthy individuals (n=27)	p-value
Gender (n, %)			P=0.957
Male	21 (63.6%)	17 (63.0%)	
Female	12 (36.3)	10 (37.0%)	
Age (years, mean±SD)	61.58±11.13	52.70±12.68	P=0.005
Hemoglobin (g/L, mean±SD)	117.79±25.33	125.59±47.19	P=0.009
Creatinine (umol/L, mean±SD)	63.69±15.48	70.67±14.69	P=0.081
Urea (mmol/L, mean±SD)	4.98±1.40	5.29±1.49	P=0.395
Blood glucose (mmol/L, mean±SD)	5.21±1.18	5.16±0.53	P=0.353

Supplementary Table 2. Clinical characteristics of the GC patients used for untargeted metabolomics.

gene	HR	HR.95L	HR.95H	pvalue
UPP1	1.360831931	1.08027011	1.714259728	0.008912236
AK5	1.344861349	1.028789416	1.758039129	0.030188605
ENTPD2	0.870614574	0.783846926	0.966986934	0.009690446
NT5E	1.240146195	1.087312493	1.414462351	0.001339277
DPYS	1.5210553	1.042302924	2.219709043	0.029644217
AK1	0.783698577	0.630616399	0.973941464	0.027942729

Supplementary Table 3. Univariate Cox regression analysis of 97 NMRGs.

	Coef	boot_SD	Coef..boot_SD
UPP1	0.327541121	0.130596641	2.508036325
AK5	0.182223238	0.220334768	0.827028976
ENTPD2	-0.098240668	0.064524876	-1.522523947
NT5E	0.145816291	0.072871783	2.000998002
DPYS	0.312228438	0.189556705	1.647150587
AK1	-0.278087858	0.129053973	-2.154818257

Supplementary Table 4. Correlation coefficients of six NMRGs.

	Coef	boot_SD	Coef..boot_SD
T cells CD8`	-1.34185683	1.26755862	-1.058615207
T cells CD4 memory activated`	-2.653592836	2.572796514	-1.031404086
Dendritic cells activated`	-3.925520195	4.568644586	-0.859230811

Supplementary Table 5. Correlation coefficients of three immune cells.
